# Randomized Controlled Trial of Modified Nasobiliary Fixation and Drainage Technique

**DOI:** 10.3389/fsurg.2022.791945

**Published:** 2022-02-23

**Authors:** Na Mi, Shuting Zhang, Zhili Zhu, Yan Yu, Wenjing Li, Lu Zheng, Lingling Chu, Jing Li

**Affiliations:** ^1^Department of Hepatobiliary Surgery, The Second Affiliated Hospital of Army Medical University, Chongqing, China; ^2^Army Medical University, Chongqing, China

**Keywords:** nasobiliary, fixed drainage technique, unplanned extubation, bile drainage, comfort, endoscopic nasobiliary drainage

## Abstract

**Objectives:**

We investigated the clinical efficacy of a modified nasobiliary fixation and drainage technique which was designed in an attempt to reduce unplanned extubation and tube blockage and improve bile drainage and the comfort of catheterized patients.

**Methods:**

From January 2019 to December 2020, 230 patients receiving Endoscopic nasobiliary drainage (ENBD) during hospitalization were recruited to this study. Participants were randomly allocated to 2 groups by using the block randomization method: in the control group: the conventional method of nasobiliary fixation was adopted after surgery; in the test group: intraoperative annular cutting of nasobiliary tubes was performed and the exposed catheter length was standardized. The modified “tube-nose-ear” three-step technique was performed after surgery. The clinical efficacy of a modified nasobiliary fixation and drainage technique was evaluated and compared between the test group and the control group.

**Results:**

The rate of unplanned extubation and incidence of complications were significantly lower in the test group than the control group. In addition, the rate of bilirubin decrease after drainage was higher in the test group. Patient discomfort during catheterization was also significantly reduced using the modified technique (*P* < 0.05).

**Conclusions:**

The modified technique of nasobiliary fixation and drainage technique can significantly reduce unplanned extubation and nasobiliary tube blockage after ENBD, facilitate biliary drainage, and improve patient comfort. This technique warrants wider application in clinical practice.

## Introduction

Endoscopic retrograde cholangiopancreatography (ERCP) is widely used in pancreatic and biliary disease treatment as it is minimally invasive, safe, effective, and can facilitates rapid rehabilitation (1–3). Endoscopic nasobiliary drainage (ENBD) (4, 5) can quickly relieve bile duct obstruction (6, 7), control infection, and cure disease (8). Nasobiliary drainage can not only effectively relieve the pressure of the biliopancreatic duct, but also provide the following benefits: ensure unobstructed drainage; reduce the chance of infection; avoid contrast agent reflux into the pancreatic duct which might cause cholangitis or pancreatitis; push away the stones embedded in the common channel or discharge them due to small friction; prevent the adverse factors induced secondary surgery owing to residual stones, residual intrahepatic bile duct stones, or pancreatic duct obstruction, injury and spasm caused by papillary edema (9). Due to the structural heterogeneity of the human nose and face, however, it is often difficult to securely and comfortably affix nasobiliary tubes, resulting in unplanned extubation (10). Unplanned extubation (UEX) refers to extubation caused intentionally by patients or by accidents; in either case, loss of intubation can seriously worsen disease prognoses (11). The estimated global rate of unplanned extubation is 2.8–20.6%, and that in China is 3.6–15.5% (12). In addition, the tube lumen is slender, and bile is viscous and prone to cholestasis, which can easily lead to inadequate drainage and increase the risk of postoperative biliary infection (13) and related complications requiring secondary operations (14). In recent years, many scholars have continuously explored and provided new methods of nasobiliary duct fixation (15), however, the irritation of the tape to the skin and the patient's comfort during catheterization were overlooked. Now, unplanned extubation and poor drainage are still common in clinical patients with nasobiliary duct. Our department previously adopteda standard fixation method, but unplanned extubation rate remained high due to catheterization discomfort and unstable adhesive tape fixation among other reasons. Therefore, a new method of nasobiliary duct fixation is needed to solve these problems. To reduce unplanned extubation rate, facilitate biliary drainage, and improve patient comfort during catheterization, our team modified the nasobiliary fixation and drainage technique for ENBD patients according to a retrospective analysis of failure cases (Patent No. ZL201420497025.2). Then we evaluated the feasibility and safety of the modified technique for clinical application.

## Methods

### Study Design and Participants

In our single centre, randomized, controlled trial, we enrolled patients receiving ENBD during hospitalization in our department from January 2019 to December 2020. **Inclusion criteria were as follows:** (1) typical clinical characteristics of biliary system disease such as abdominal pain, jaundice, and vomiting, and receiving ERCP+ENBD after diagnosis by magnetic resonance imaging (MRI); (2) 18–65 years old; (3) inpatient with nasobiliary tube indwelling after ERCP; (4) informed consent to drainage and intervention; (5) ability to cooperate closely with postoperative treatment and evaluation. **Exclusion criteria were as follows:** (1) pregnant, breast feeding, or with diagnosed mental disorders; (2) operation failure; (3) inability to communicate normally; (4) preoperative abdominal pain, cholangitis, pancreatitis, increased body temperature, elevated white blood cell count, or increased serum amylase; (5) abnormal coagulation function, liver function de-compensation in combination with cardiovascular, cerebrovascular, or kidney disease, intestinal stenosis or intestinal obstruction, or moderate to severe esophageal or gastric fundus varices. Patients who withdraw due to irresistible factors or serious complications not suitable for continuing the study after being confirmed by a competent physician. Participants provided written, informed consent before randomization. This study was approved by the institutional ethics committee of Hospital.

### Randomization

Participants were randomly assigned using a blocked randomization scheme generated by a computer (block size four based on a random table from an independent statistician), grouped by the modified method of nasobiliary fixation and conventional method of nasobiliary fixation (control group) in a 1:1 ratio.

According to a preliminary experiment we carried out, the unplanned extubation rate in the modified method of nasobiliary fixation group was 18%, whereas that of the conventional method of nasobiliary fixation was 5%. Allowing for 10% loss of follow up, we needed to at least recruit 100 participants in each group to ensure an experiment with 80% power and 5% significance level to account for the two comparisons.

**Figure 1:**
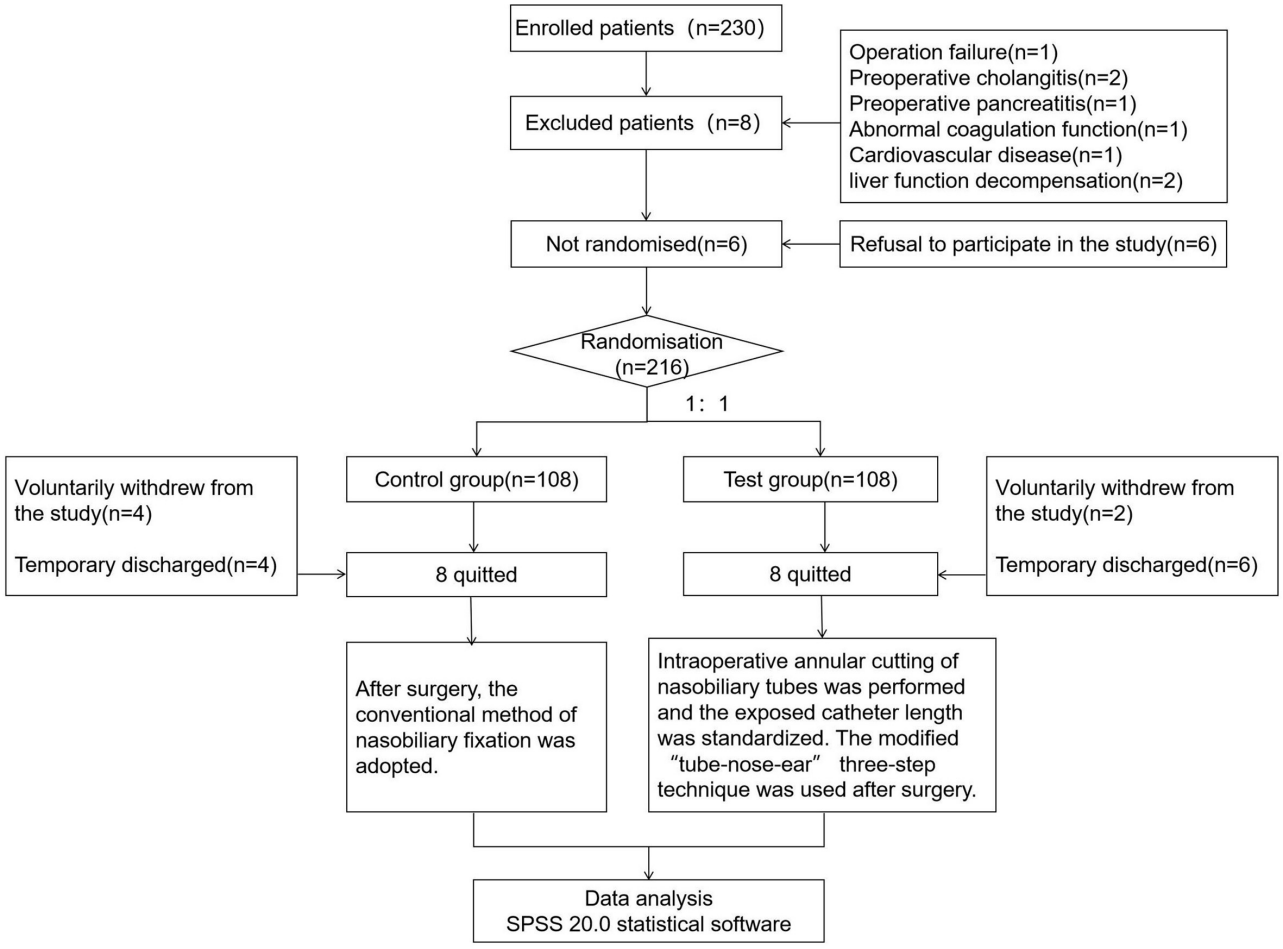
Flowchart of the study design.

Two hundred and thirty patients who received ENBD during hospitalization in our department were recruited to this study. Among the 230 participants, 14 were excluded [operation failure (*n* = 1), preoperative cholangitis (*n* = 2), preoperative pancreatitis (*n* = 1), abnormal coagulation function (*n* = 1), cardiovascular disease (*n* = 1), liver function de-compensation (*n* = 2), refusal to participate in the study (*n* = 8)], 16 quitted (8 in the control group and 8 in the test groups). Six patients voluntarily withdrew from the study (4 in the control group and 2 in the test groups), and 10 participants were discharged (4 in the control group and 6 in the test groups) (Figure 1).

### Procedures

Patients and families were informed of the importance and precautions necessary to maintain indwelling nasobiliary tubes. ERCP+ENBD was performed according to the conventional method. After the tube was successfully inserted, catheterization depth, exposed length, and indwelling duration were recorded. Comprehensive nursing was performed after these operations (refer to Comprehensive Nursing of Nasobiliary Drainage after ERCP) (16).

**Figure 2:**
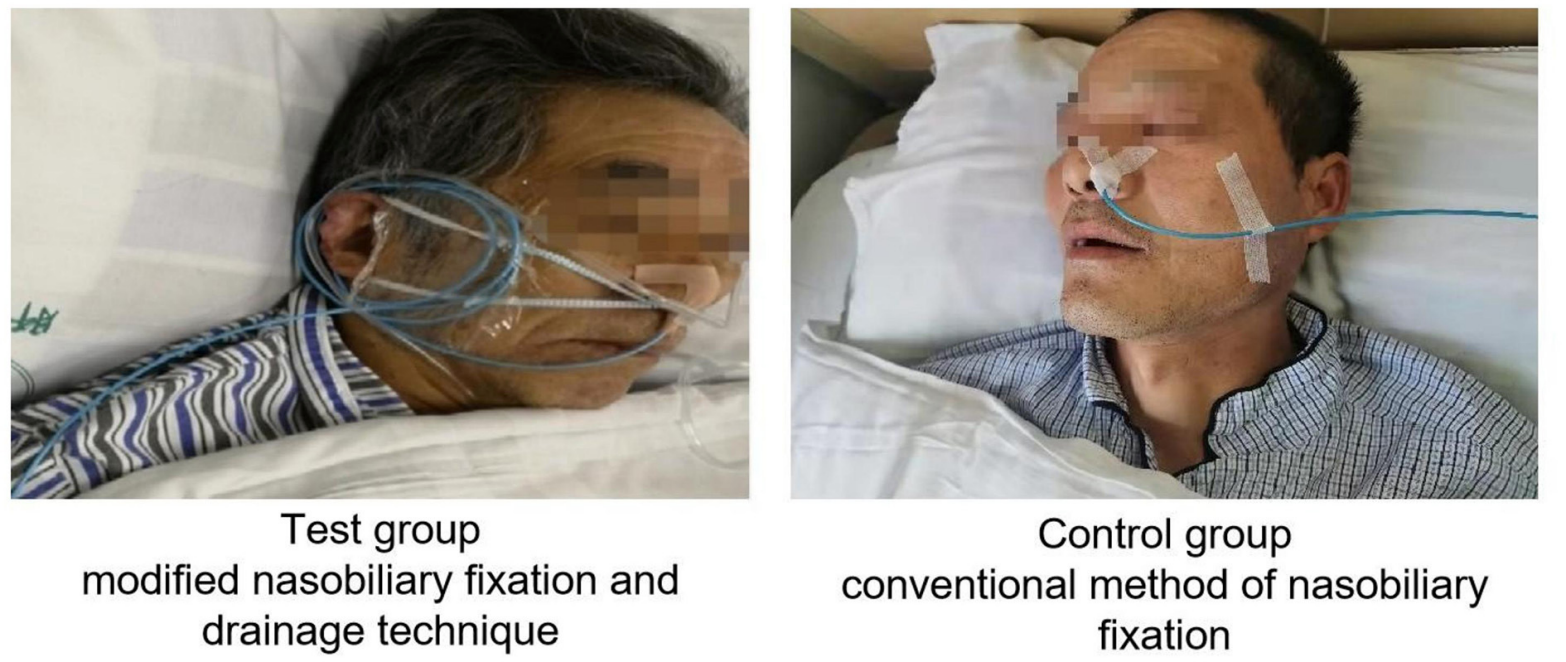
Two groups of the nasobiliary fixation technique.

In the control group: after surgery, the conventional method of nasobiliary fixation was adopted. Two strips of medical adhesive tape were used to fix a nasobiliary tube. One strip was overlapped to fix the nasobiliary tube onto the ala nasi and the other strip was lifted to fix the nasobiliary tube on the ipsilateral cheek. The nasobiliary tube was fixed once more from the bottom up around the ipsilateral ear. In the test group: intraoperative annular cutting of nasobiliary tubes was performed and the exposed catheter length was standardized. The modified “tube-nose-ear” three-step technique was performed after surgery (Figure 2).

#### Modified Nasobiliary Fixation and Drainage Technique

(1) Modified materials.

Elastic adhesive tape from 3M was selected as the fixation material due to better viscosity, water resistance, air permeability, and extensibility than traditional medical adhesive tape (17). It can also be cut to fit each patient's nose features. Additionally, it is inexpensive and allows longer application time.

(2) Modified “tube-nose-ear” three-step fixation of nasobiliary tubes.

**Figure 3:**
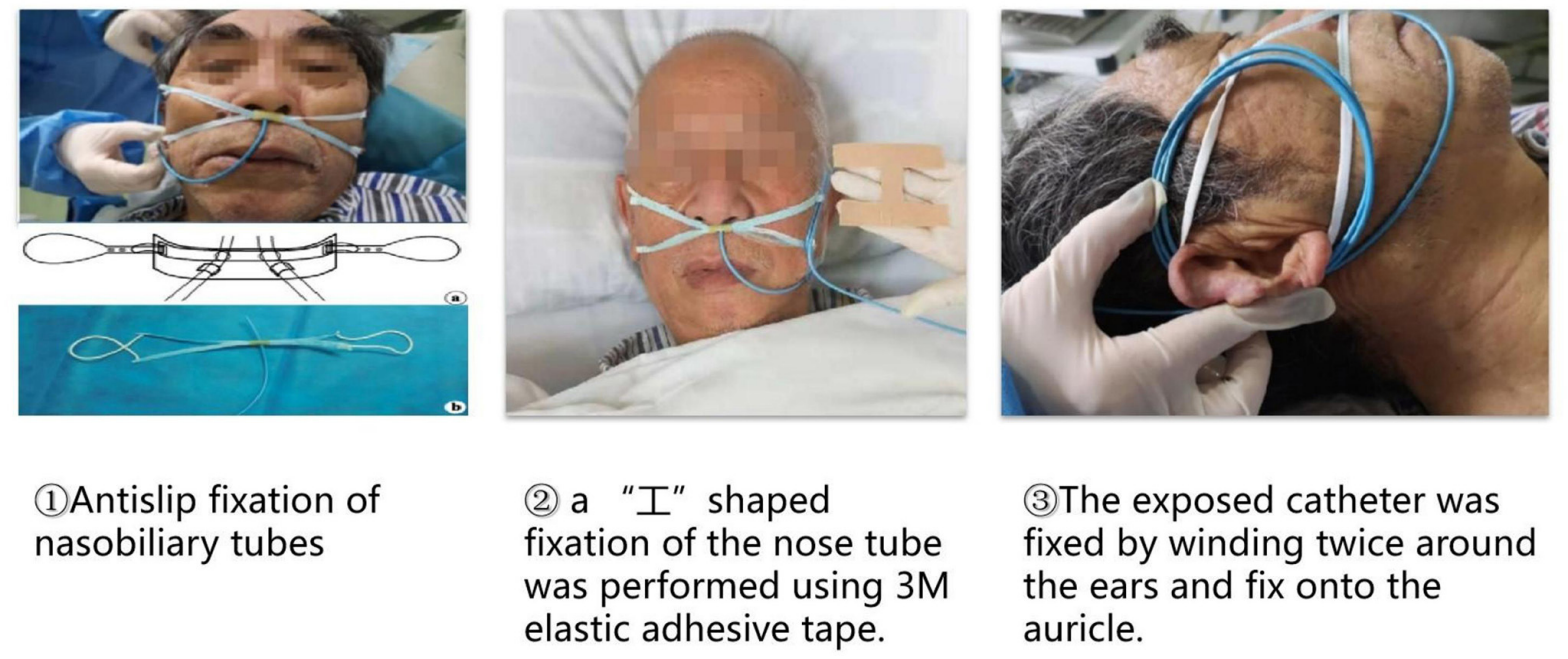
Modified “tube- nose-ear” three-step fixation of nasobiliary tubes.

First, we developed a method for anti-slip fixation of nasobiliary tubes. A short section of rubber tubing about 1 cm long was cut and two holes about 0.1 cm in diameter were punched through the middle (i.e., each 0.5 cm from the ends and on opposite sides of the tube). The mid-section of a disposable surgical mask was inserted up to (but not blocking) the two middle holes and the nasobiliary tube passed through the two holes. This “+” structure was then hung on both ears by the mask strings. Second, “I” shape fixation of the nose tube was performed using 3M elastic adhesive tape. Transparent dressing (3M) was pasted on one cheek for double fixation. Third, the exposed catheter was fixed by winding twice around the ears and fixed onto the auricle. In this conformation, the catheter on the cheek was at about 11-cm long, and the angle between the tube and the ala nasi was about 5° (Figure 3).

(3) Modified technique for measuring and cutting nasobiliary tubes.

**Figure 4:**
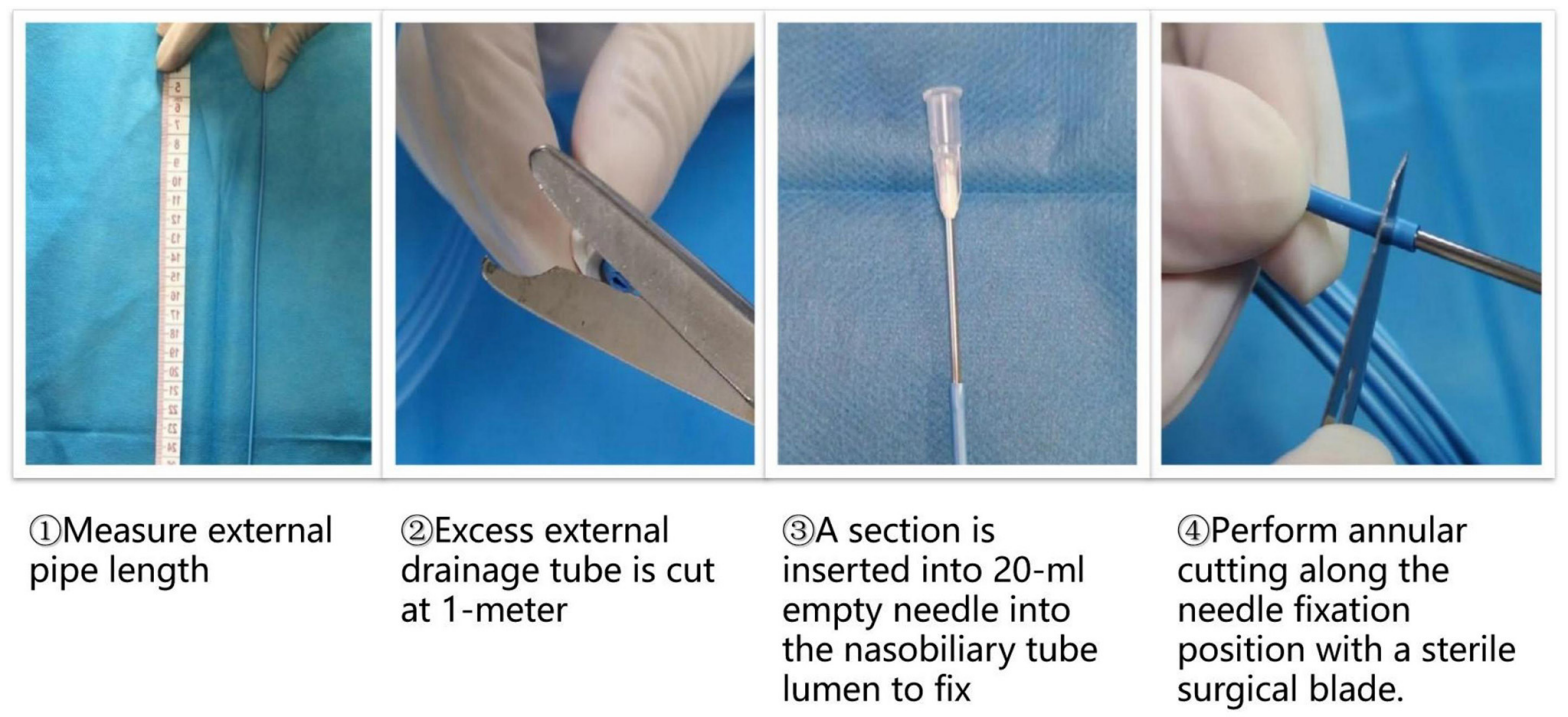
Modified technique for measuring and cutting nasobiliary tubes.

For annular cutting of the tube, a section was inserted into 20-ml empty needle into the nasobiliary tube lumen to fix and perform annular cutting along the needle fixation position with a sterile surgical blade. Second, to standardize the external drainage tube length, excess external drainage tube was routinely cut at 1-meter after surgery (Figure 4).

### Outcomes

The primary outcome of efficacy was unplanned extubation rate of nasobiliary tubes, Secondary outcomes of efficacy was nasobiliary tube blockage rate. For safety, serum bilirubin reduction rate after drainage, incidence of postoperative complications, frequency of local skin irritation, and discomfort from nasobiliary tubes were compared between modified and conventional method of nasobiliary fixation groups. In addition, physiological discomfort was assessed.

The questionnaire on physiological discomfort from nasobiliary tubes developed by Wu Q was adopted (18). This assessment includes 7 items: nasal cavity discomfort, nausea and vomiting, throat discomfort, dry mouth, abdominal discomfort, limited neck movement, and sleep disorders, each of which were evaluated on a visual analog scale (VAS) (19), with a maximum total score of 10. A score of 0–2 indicated scomfort, 3–4 points mild discomfort, 5–6 points moderate discomfort, 7–8 points severe discomfort, and 9–10 points extreme discomfort.

### Statistical Analysis

Data acquisition personnel were uniformly trained, methods were standardized, and a database was established and maintained by specified personnel. All enrolled subjects were surveyed by questionnaires issued by researchers. When questionnaires were returned, researchers checked whether there were any omissions. If so, the missing items were filled out on the spot. The questionnaires effective recovery rate was 100%. All data were analyzed using the statistical software SPSS 20.0 (IBM Corp, Armonk, NY, USA). Continuous variables were expressed as mean ± standard deviation and categorical variables as frequency and ratio. Group means were compared by independent sample *t*-test and ratios by chi-square (χ^2^) test. *P* < 0.05 was considered statistically significant.

## Results

### Patient Demographics and Baseline Clinical Variables

A total of 200 patients were included, 100 each in the test and control groups. There were no statistically significant differences in age, sex ratio, disease, education level and body mass index(BMI) between groups (*P* > 0.05) (Table 1).

**Table 1:** Comparison of general data in patients [*n* (%)].

	**Groups**		**χ^2^/t**	***P***
	**Control group**	**Test group**		
	**(*n* = 100)**	**(*n* = 100)**		
**Gender**				
Male	44 (44%)	57 (57%)	3.38	0.066
Female	56 (56%)	43 (43%)		
**Age (years)**	48.79 ± 1.28	48.19 ± 1.84	0.27	0.788
**BMI (kg/m^2^)**	22.79 ± 0.48	23.64 ± 0.40	1.36	0.174
**Disease**			3.722	0.881
Obstructive jaundice	4 (4%)	3 (3%)		
Choledocholithiasis	60 (60%)	63 (63%)		
Cholangiolithiasis	17 (17%)	15 (15%)		
Bile leakage	1 (1%)	0 (0%)		
Cholangiocarcinoma	0 (0%)	1 (1%)		
Cholangiectasis	5 (5%)	7 (7%)		
Cholecystolithiasis	11 (11%)	10 (10%)		
Pancreatolithiasis	1 (1%)	0 (0%)		
Biliary stricture	1 (1%)	1 (1%)		
**Education level**			1.899	0.594
Primary school	39 (39%)	43 (43%)		
Junior school	32 (32%)	26 (26%)		
Senior school	18 (18%)	23 (23%)		
Higher education	11 (11%)	8 (8%)		

*Values are mean ± standard deviation or number*.

*P-values were calculated by χ^2^ test or independent samples t-test*.

### Comparison of Unplanned Nasobiliary Tube Extubation Rates, Blockage Rates, Indwelling Catheters Time, Reasons of Unplanned Extubation and Inadequate Drainage Between Test and Control Groups

The total incidence of postoperative unplanned extubation was significantly lower in the test group than the control group (5 vs. 14%, *P* < 0.05). The total incidence of postoperative nasobiliary tube blockage was also significantly lower in the test group (7 vs. 18%, *P* < 0.05). The indwelling catheters time in experimental group was significantly higher than that in control group (*P* < 0.05) (Tables 2–4).

**Table 2:** Comparison of unplanned extubation, inadequate drainage rates and indwelling catheters time between test and control group patients [*n* (%)].

**Items**	**Control group (*n* = 100)**		**Test group (*n* = 100)**		**χ^2^/t**	***P***
	**Yes**	**No**	**Yes**	**No**		
Unplanned extubation	14 (14.0%)	86 (86.0%)	5 (5.0%)	95 (95.0%)	4.71	**0.030**
Inadequate drainage	18 (18.0%)	82 (82.0%)	7 (7.0%)	93 (93.0%)	5.53	**0.019**
Indwelling catheters time	2.44 ± 0.153		3.58 ± 0.233		4.085	**0.000**

**Table 3:** Reasons of unplanned extubation between test and control group patients [*n* (%)].

**Reasons**	**Control group**	**Test group**
	**(*n* = 14)**	**(*n* = 5)**
Special nasofacial structures causing unstable fixation	4 (28.6%)	0 (0%)
Adhesive tape loosened easily	2 (14.3%)	0 (0%)
Local skin irritation was not tolerated by patients	2 (14.3%)	1 (20%)
Catheterization caused discomfort	2 (14.3%)	2 (40%)
Exposed catheters were too long and affected patient movement	2 (14.3%)	0 (0%)
Patients and family members paid insufficient attention	1 (7.1%)	1 (20%)
Patients had dysphoria or delirium and were not restrained on time	1 (7.1%)	1 (20%)

**Table 4:** Reasons for inadequate drainage between test and control group patients [*n* (%)].

**Reasons**	**Control group**	**Test group**
	**(*n* = 18)**	**(*n* = 7)**
Nasobiliary tube lumen was slender	2 (11.1%)	1 (14.2%)
Viscous bile clogged easily	3 (16.7%)	3 (42.9%)
Exposed catheters of nasobiliary tubes were too long and coils folded easily	5 (27.8%)	0 (0%)
Junctions were too tight and deformed/compressed tubes	6 (33.3%)	0 (0%)
Silt-like calculi and purulent flocon structube lumen	2 (11.1%)	3 (42.9%)

### Comparison of Postoperative Complications and Unobstructed Drainage During Nasobiliary Tube Indwelling Between Two Groups of Patients

The total incidence of postoperative hyperamylasemia was significantly lower in the test group than the control group (2 vs. 10%, *P* < 0.05). Similarly, total incidence of postoperative biliary infection was lower in the test group (1 vs. 7%, *P* < 0.05). The bilirubin reduction rate in experimental group was significantly higher than that in control group (*P* < 0.05) (Table 5).

**Table 5:** Comparison of postoperative complications between test and control groups [*n* (%)].

**Complications**	**Control group (*n* = 100)**		**Test group (*n* = 100)**		**χ^2^/t**	***P***
	**Yes**	**No**	**Yes**	**No**		
Hyperamylasemia	10 (10.0%)	90 (90.0%)	2 (2.0%)	98 (98.0%)	5.67	**0.017**
Biliary tract infection	7 (7.0%)	93 (93.0%)	1 (1.0%)	99 (99.0%)	4.69	**0.030**
Pancreatitis	18 (18.0%)	82 (82.0%)	10 (10.0%)	90 (90.0%)	2.66	0.103
Bilirubin reduction rate	5.92 ± 4.61 (6 ± 5%)		61.39 ± 9.16 (32 ± 4%)		3.87	**< 0.001**

*Values are mean ± standard deviation or number (percentage)*.

*P-values were calculated by χ^2^ test or independent samples t test, and bold represents significant differences among subgroups*.

### Comparison of Physiological Discomfort and Local Skin Irritation Rates Between Test and Control Groups

During nasobiliary tube indwelling, the proportion of patients reporting no substantial discomfort was significantly higher in the test group than the control group (19 vs. 9%, *P* < 0.05). The proportion reporting only mild discomfort was also significantly higher in the test group (37 vs. 23%, *P* < 0.05), while the proportion reporting severe/extreme discomfort was significantly lower in the test group (10 vs. 21%, *P* < 0.05). The rate of local skin irritation during catheterization was also lower in the test group (8 vs. 18%, *P* < 0.05) (Table 6).

**Table 6:** Comparison of discomfort level and skin changes between test and control groups [*n* (%)].

**Items**	**Control group (*n* = 100)**		**Test group (*n* = 100)**		**χ^2^**	***P***
	**Yes**	**No**	**Yes**	**No**		
**Discomfort level**						
Comfort	9 (9.0%)	91 (91.0%)	19 (19.0%)	81 (81.0%)	4.15	**0.042**
Mild discomfort	23 (23.0%)	77 (77.0%)	37(37.0%)	63 (63.0%)	4.67	**0.031**
Moderate discomfort	47 (47.0%)	53 (53.0%)	34 (34.0%)	64 (64.0%)	3.51	0.061
Severe/extreme discomfort	21 (21.0%)	79 (79.0%)	10 (10.0%)	90 (90.0%)	4.62	**0.032**
Skin changes	18 (18.0%)	82 (82.0%)	8 (8.0%)	92 (92.0%)	4.42	**0.036**

*Values are number (percentage). P-values were calculated by χ^2^ test, and bold represents significant differences among subgroups*.

## Discussion

### Clinical Efficacy of the Modified “Tube-Nose-Ear” Three-Step Fixation Technique for Nasobiliary Tubes

Our research group modified the nasobiliary fixation and drainage technique according to the primary reasons for unplanned extubation documented in previous cases, and then assessed the efficacy of this new “tube-nose-ear” three-step fixation method in a controlled clinical trial. Unplanned extubation rate and catheter coil folding rate were indeed significantly reduced using the “tube-nose-ear” three-step fixation technique compared to the standard control method. The reasons for this improved efficacy were as follows. (1) The anti-slip nasobiliary fixation technique effectively secured the catheter outside the opening of the nasal meatus to prevent displacement. (2) 3M adhesive tape and dressing were used in the nose for “I” shape double fixation of the nasofacial tube. Liquid dressing was also used to protect sensitive skin in advance to increase stability and comfort. (3) Winding the exposed catheters around the ears reduced bending and folding of the nasobiliary tube and allowed seamless adhesion to the face. In this conformation, the nasobiliary tube was difficult to pull out. Simultaneously, physiological comfort was significantly greater in the test group than the control group, which may be related to more stable tube fixation, thereby reducing stimulation of the larynx while minimizing hindrance on patient movements. Therefore, the modified “tube-nose-ear” three-step fixation technique can effectively reduce the rate of unplanned extubation and improve patient comfort without requiring extensive training, cleaning, or disinfection. This technique thus warrants wider clinical application.

### The Advantages of 3M Elastic Adhesive Tape and Transparent Dressing for Fixing Nasobiliary Tubes

By analyzing the primary reasons for unplanned extubation in previous patients, we found that factors related to conventional medical adhesive tape accounted for 54% of all incidences. Therefore, we switched from conventional medical adhesive tape to 3M elastic adhesive tape in the test group and observed significantly reduced unplanned extubation rate. The poor fixation of the conventional medical adhesive tape was mainly due to its low viscosity and insufficient extensibility, resulting in easily loosening during patient activities and by skin oil secretions and perspiration. Longer indwelling time also increased the risk of unplanned extubation. Therefore, in the modified technique we applied: (1) the use of 3M elastic adhesive tape, which contains elastic fiber and can be cut to the appropriate length according to naso-facial structure, (2) double fixation of tubes on the cheek with 3M transparent dressing. The stable viscosity of 3M dressing allowed close attachment to the skin and catheter, and the transparency allows for easy confirmation of secure fixation (16, 20). The usage of 3M elastic adhesive tape and 3M transparent dressing also alleviated local skin irritation, as proved by the lower number of extubation cases caused by skin allergy in the test group; However, the difference did not reach statistical significance. This lower rate of irritation may stem from the hypoallergenic nature of 3M medical adhesive. In addition to the adhesive tape, individual allergic constitution and therapeutic drug hypersensitivity may contribute to local skin allergy, which needs further examination.

### The Modified Technique for Measuring and Cutting Nasobiliary Tubes Can Promote Biliary Drainage and Reduce Complications

The effective biliary drainage rate using the conventional technique is limited by frequent tube blockage (21). In our department, the rate of nasobiliary tube blockage was even lower (60.8%) using the conventional technique than in previous studies (76.67%). Effective biliary drainage can alleviate symptoms of biliary obstruction, jaundice, skin pruritus and fever, relieve the purpose of biliary pressure, improve patient liver function, and facilitate disease recovery (2). No previous studies have examined if measuring and cutting nasobiliary tubes can improve biliary obstruction rate. The effect of nasobiliary drainage as determined by the decrease in serum bilirubin on the 3rd day post-operation compared to pre-surgery (22) was significantly higher in the test group. Possible reasons for this improvement are (1) the removal of excess external drainage tube for a standardizing length and (2) the winding and cutting technique ensuring that the lumen would not be deformed, thereby avoiding an unsecure tube connection. Thus, this modified technique was more conducive to biliary drainage, did not affect postoperative retreatment and angiography, and prevented the occurrence of complications. The most frequent postoperative ENBD complications were biliary infection, pancreatitis, and hyperamylasemia (23). The incidences of hyperamylasemia and biliary infection were significantly lower in the test group than the control group. The incidence of pancreatitis was also lower in the test group, although the difference did not reach statistical significance level. It was possible that the use of contrast agents during patient examination and treatment was a potential cause of pancreatitis (24), a question that warrants further study. Nonetheless, our modified technique of measuring and cutting nasobiliary tubes can facilitate biliary drainage, reduce complications, and promote rapid recovery with excellent clinical practicability.

## Conclusions

Compared to the conventional nasobiliary fixation and drainage approach, our modified “tube-nose-ear” three-step fixation method significantly reduced unplanned extubation and complication rates, and improved patient comfort. Moreover, this technique was relatively simple to perform and cost-effective, so it could be widely applied in clinical practice.

## Data Availability Statement

The raw data supporting the conclusions of this article will be made available by the authors, without undue reservation.

## Ethics Statement

The studies involving human participants were reviewed and approved by the institutional Ethics Committee of Xinqiao Hospital of Army Medical University (2019-065-01). Written informed consent for participation was not required for this study in accordance with the national legislation and the institutional requirements.

## Author Contributions

JL: conception and design. LC: administrative support. NM: provision of study materials or patients. NM, LZ, SZ, ZZ, YY, and WL: collection and assembly of data. All authors data analysis and interpretation, manuscript writing, and final approval of manuscript.

## Funding

This work was supported by the Army Medical University for the second level of clinical new technology (2018-2-2-43), the Natural Science Foundation of Chongqing (cstc2021jcyj-msxm3464), and Clinical research project of the Second Affiliated Hospital of Army Military Medical University (XQQCC-2015-004).

## Author Disclaimer

All claims expressed in this article are solely those of the authors and do not necessarily represent those of their affiliated organizations, or those of the publisher, the editors and the reviewers. Any product that may be evaluated in this article, or claim that may be made by its manufacturer, is not guaranteed or endorsed by the publisher.

## Conflict of Interest

The authors declare that the research was conducted in the absence of any commercial or financial relationships that could be construed as a potential conflict of interest.

## Publisher's Note

All claims expressed in this article are solely those of the authors and do not necessarily represent those of their affiliated organizations, or those of the publisher, the editors and the reviewers. Any product that may be evaluated in this article, or claim that may be made by its manufacturer, is not guaranteed or endorsed by the publisher.
