# Supplementary material for: Randomized Controlled Trial of Modified Nasobiliary Fixation and Drainage Technique
Source: Front Surg. 2022 Feb 23;9:791945. doi: 10.3389/fsurg.2022.791945 (PMC8904870; doi:10.3389/fsurg.2022.791945)
Supplement: Supplementary file 1 [file Presentation_1.PPTX]

## Slide 1
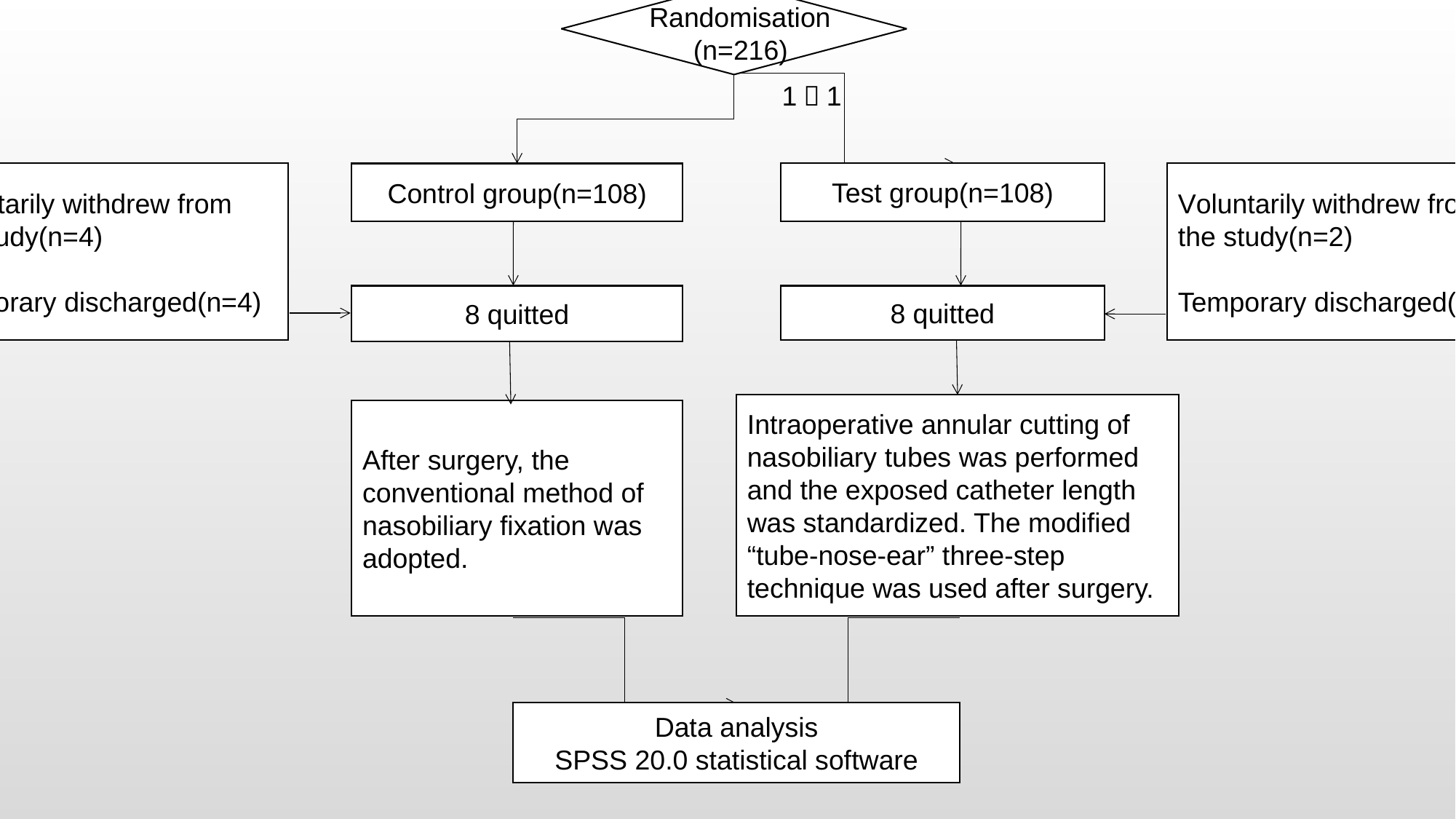

Enrolled patients（n=230）
Operation failure(n=1)
Preoperative cholangitis(n=2)
Preoperative pancreatitis(n=1)
Abnormal coagulation function(n=1)
Cardiovascular disease(n=1)
liver function decompensation(n=2)
 Excluded patients（n=8）
Not randomised(n=6)
Refusal to participate in the study(n=6)
Randomisation
(n=216)
1：1
Voluntarily withdrew from the study(n=4)
Temporary discharged(n=4)
Control group(n=108)
Test group(n=108)
Voluntarily withdrew from the study(n=2)
Temporary discharged(n=6)
8 quitted
8 quitted
Intraoperative annular cutting of nasobiliary tubes was performed and the exposed catheter length was standardized. The modified “tube-nose-ear” three-step technique was used after surgery.
After surgery, the conventional method of nasobiliary fixation was adopted.
Data analysis
SPSS 20.0 statistical software
Figure 1 Flowchat of the study design

## Slide 2
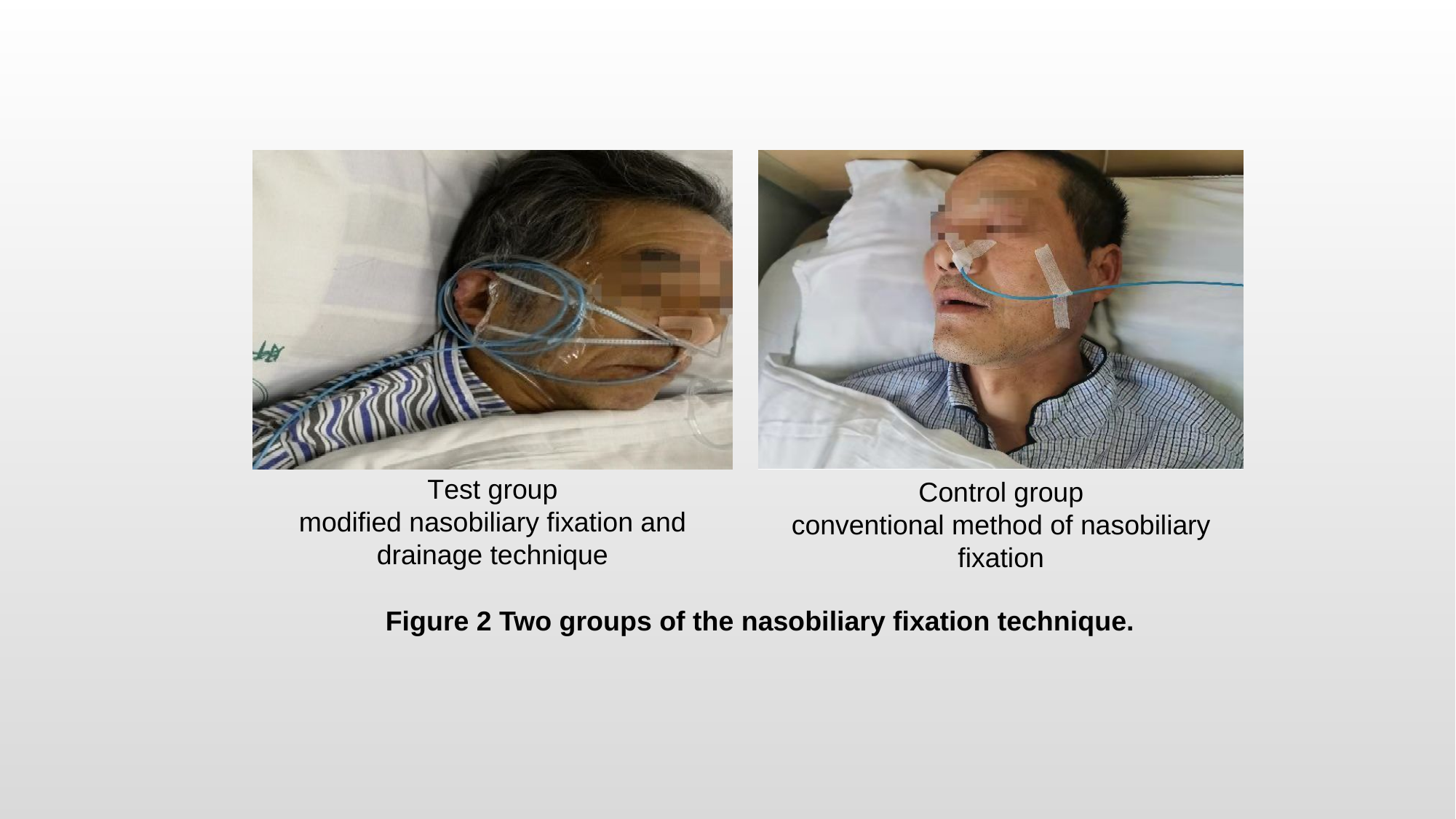

Test group
modified nasobiliary fixation and drainage technique
Control group
conventional method of nasobiliary fixation
Figure 2 Two groups of the nasobiliary fixation technique.

## Slide 3
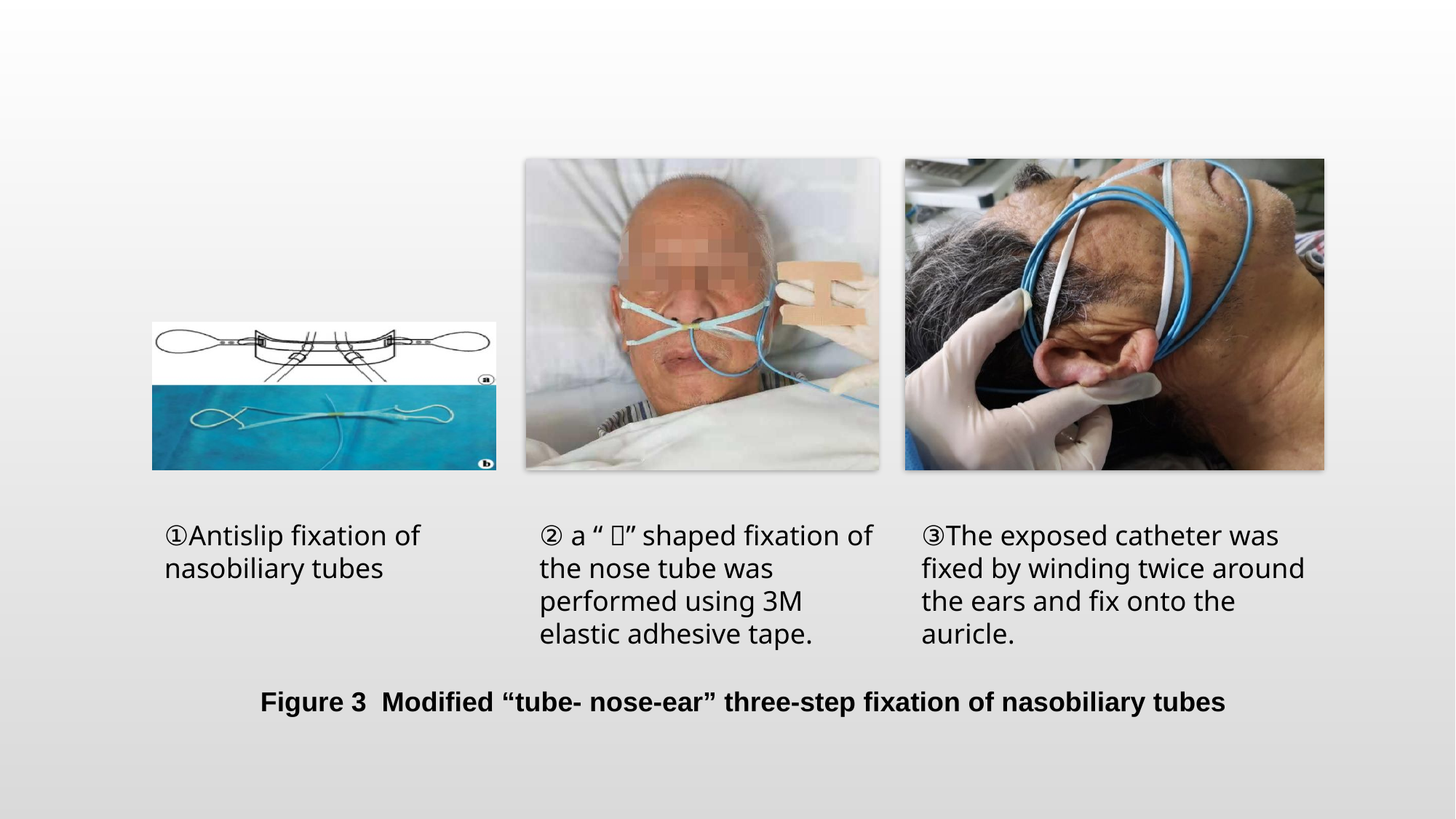

①Antislip fixation of nasobiliary tubes
② a “工”shaped fixation of the nose tube was performed using 3M elastic adhesive tape.
③The exposed catheter was fixed by winding twice around the ears and fix onto the auricle.
Figure 3 Modified “tube- nose-ear” three-step fixation of nasobiliary tubes

## Slide 4
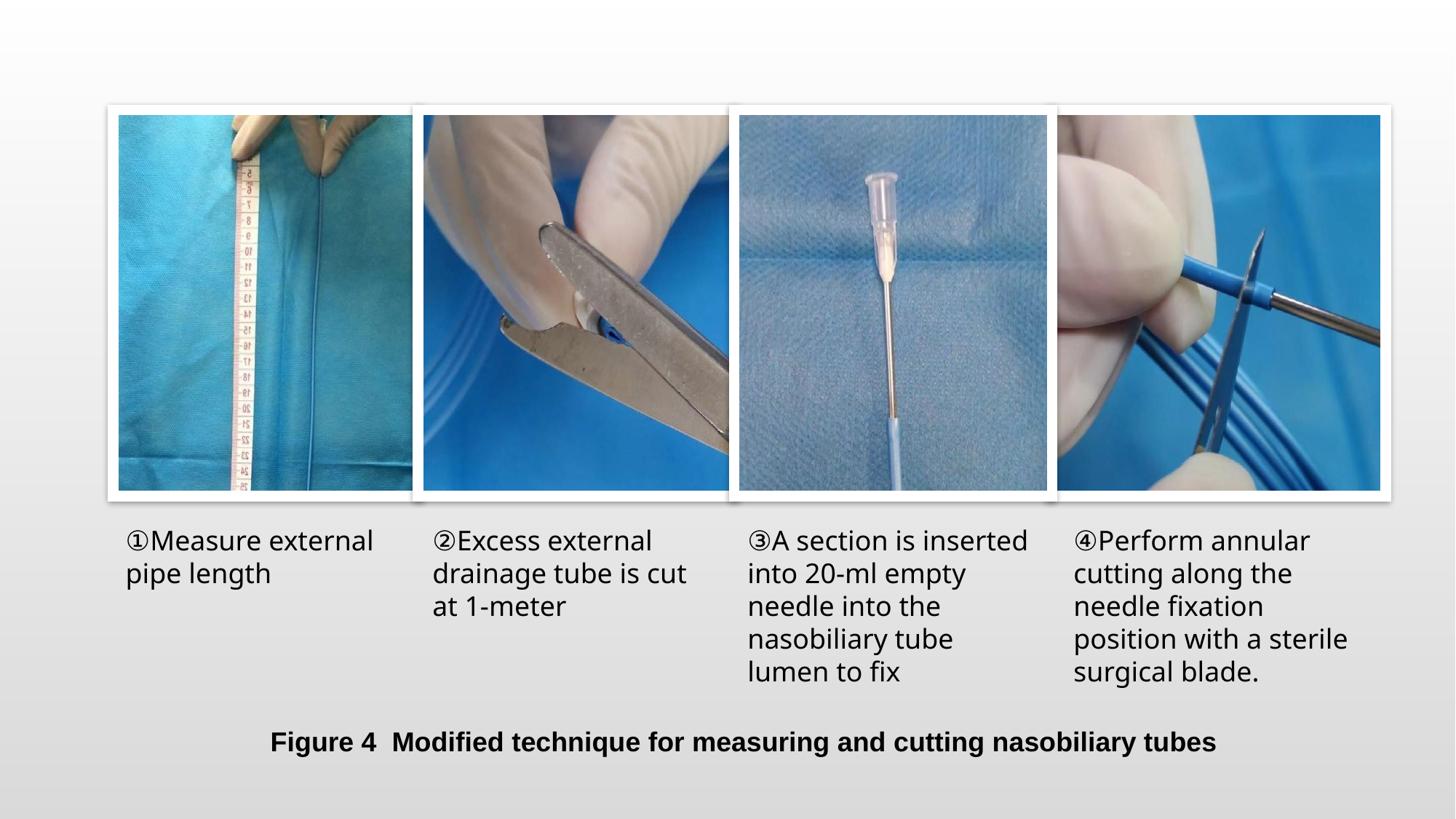

①Measure external pipe length
②Excess external drainage tube is cut at 1-meter
③A section is inserted into 20-ml empty needle into the nasobiliary tube lumen to fix
④Perform annular cutting along the needle fixation position with a sterile surgical blade.
Figure 4 Modified technique for measuring and cutting nasobiliary tubes
